# Child and adolescent mental health services in the Western Cape Province of South Africa: the perspectives of service providers

**DOI:** 10.1186/s13034-022-00491-w

**Published:** 2022-07-14

**Authors:** Stella Mokitimi, Kim Jonas, Marguerite Schneider, Petrus J. de Vries

**Affiliations:** 1 Division of Child and Adolescent Psychiatry, University of Cape Town, 46 Sawkins Road, Rondebosch, Cape Town, 7700 South Africa; 2 South African Medical Research Council, Cape Town, South Africa; 3 Alan J Flisher Centre for Public Mental Health, University of Cape Town, Cape Town, South Africa

**Keywords:** Child and adolescent mental health, Health systems, Western Cape, South Africa, Perspectives, Service providers, Low- and middle-income countries

## Abstract

**Background:**

Current work in the field point to the need to strengthen child and adolescent mental health services (CAMHS) globally, and especially in low- and middle-income countries (LMICs). Policy development, planning and service provision must be relevant to the needs of stakeholders at grassroots level, and should include their perspectives. This study set out to explore the perspectives and lived experiences of service providers, including their recommendations to strengthen CAMHS in South Africa.

**Methods:**

Using focus group discussions (FGDs) and semi-structured individual interviews (SSIIs), qualitative data were collected from 46 purposefully selected multidisciplinary health service providers across the Western Cape, one of the nine provinces of South Africa. Audio-recorded data were entered into NVivo 11 (QSR), and thematic analysis was performed by two independent raters.

**Results:**

Results highlighted a significant lack of CAMH resources, poor intersectoral collaboration, limited access to training, absence of consistency and uniformity in service delivery, weak support for staff, and high rates of negative attitudes of staff. External factors contributing to poor CAMHS identified by service providers included poor socioeconomic circumstances, high rates of HIV/AIDS, substance use and stigma. The eight recommendations to strengthen CAMHS included a need to (1) increase CAMH staffing, (2) provide dedicated CAMHS at secondary care and child-friendly infrastructure at primary care, (3) review current service focus on number of patients seen versus quality of care provided to children, (4) formalise intersectoral collaborations, (5) increase learning opportunities for trainees, (6) employ a lead professional for CAMHS in the province, (7) increase support for staff, and (8) acknowledge staff initiatives.

**Conclusions:**

Findings underlined the need for quality improvement, standardisation and scale-up of mental health services for children and adolescents in South Africa. Whilst we used the Western Cape as a ‘case study’, we propose that our findings may also be relevant to other LMICs. We recommend that the perspectives of service users, including children and adolescents, be sought to inform service transformation.

## Background

There is an urgent need to recognise child and adolescent mental health (CAMH) problems as a public health priority, and to give this priority the attention it deserves. Across the globe approximately one in five children and adolescents suffer from one or more mental illness [1, 2], thus representing a major cause of morbidity [3]. The evidence-base for the burden of child and adolescent mental disorders in low- and middle-income countries (LMICs) is particularly limited [1]. Insufficiently skilled human resources, low awareness and low priority, high service load, greater concern for child mortality than morbidity, and journal acceptance biases against LMICs research may all contribute to this small evidence-base [1, 4]. The little evidence available shows that poverty and parental unemployment are contextual risk factors for poor CAMH and for developing child and adolescent mental disorders. Brain injuries, consequent neuropsychiatric morbidity, intellectual disability and epilepsy are more common in LMICs than in high-income countries, and these disorders have a significant impact on educational attainment [4] and on the likelihood of other mental health disorders occurring.

To strengthen health systems such as CAMH systems, all the elements of a particular health system (referred to as the ‘hardware’ and ‘software’) should be evaluated [5, 6]. This requires an in-depth exploration of multiple levels of CAMH services and systems (CAMHSS) including the policy, resource, senior stakeholder, provider, and user landscapes [7]. In a policy analysis of CAMH in South Africa, findings showed a clear neglect at policy level in all nine of the provinces, including the Western Cape, the province under investigation here [8]. A National Child and Adolescent Mental Health policy (2003) stated that CAMH services should be provided at all three levels of care (primary, secondary and tertiary), outlined the movement of children between these tiers and the types of assessment and intervention to be offered. While a national CAMH policy existed to guide the development and implementation of CAMH policies in individual provinces, none of the provinces developed provincial CAMH policies or implementation plans to give effect to the national policy. These findings were concerning, given that good service provision should start with good policies. A fine-grained situational analysis of the resource landscape for CAMHSS in the Western Cape [9] identified significant gaps across all ‘hardware’ elements (policies, funding, clinical services, primary healthcare, human resources, intersectoral collaboration, and information systems), and software elements (e.g. training in CAMH). The limited training in CAMH varied across levels of care. At primary care level, training was provided in the form of outreach and support, workshops and seminars, and clinical consultation provided by CAMH specialist teams to primary care staff on an ad hoc basis. No dedicated training on CAMH was provided to level 2 teams. At tertiary level, the province had only 2 funded specialist training posts in child & adolescent psychiatry, and no funded training programmes for psychologists, nurses, occupational therapists or speech and language therapists in CAMHS. Staff with joint appointments (employed by the university and by the provincial government) had a very small percentage (< 30%) of their time allocated for training, teaching and/or research, and staff appointed by the Western Cape Government had little to no time allocated for training, supervision and/or research.

To examine the senior stakeholder landscape, an analysis of Strenghts, Weaknesses, Opportunities and Threats for CAMHS (SWOT analysis) was performed with a group of key senior stakeholders in the province (funders, policymakers, senior service managers, and senior clinicians) [7]. This approach allowed us to collect information not only about hardware elements, but also about some of the ‘software’ elements of our CAMH systems, such as beliefs, attitudes, relationships, and values [6]. The findings of the SWOT analysis identified many weaknesses and threats to CAMH in the province, in line with the observations made in the situational analysis. The SWOT analysis also identified areas of strengths and opportunities that could be used to support service strengthening in CAMHS. We proposed a ‘tipping point’ model [7] suggesting that a number of positive and negative forces interact on the scale of services. The scale was predominantly skewed towards negative tipping points. In a tipping point model, service strengthening could be achieved not only through major restructuring, but also through the collective effects of small positive tipping events.

In the Western Cape, the Department of Health launched the Desmond Tutu youth-friendly initiative in partnership with the Desmond Tutu HIV Foundation. The initiative created services to young people between 10 and 24 years of age to make healthcare services more accessible to the needs of youth, to keep young girls in school, and to decrease the rates of teenage pregnancy, Tuberculosis (TB), and HIV/AIDS. While this was a very positive initiative, it did not include any elements related CAMH.

This study set out to explore the perceptions of service providers about CAMHSS in the Western Cape and to seek their recommendations for future strategies to strengthen services. We selected to focus on service providers for two reasons: Firstly, grassroots level service provider perspectives are important to help gain insight into the real-world context in which services are provided. In the Western Cape there has been very limited exploration of the views of CAMH service providers at grassroots level who have direct day-to-day clinical interactions with children, adolescents and their families. Secondly, these perspectives may be able to provide valuable practical observations that could suggest further tipping points to be used for the strengthening of CAMHSS. This study was designed to follow on the earlier policy and SWOT analyses performed with senior stakeholders (including policy-makers, funders, provincial leads and service managers) to ensure that we can generate a multi-level analysis of needs, perspectives and recommendations to inform service strengthening in CAMHS. We therefore set out to collect qualitative data from a broad range of service providers across the urban and rural geographical service areas in the province, and across all levels of care (primary, secondary and tertiary). We were interested to see if we would be able to identify a) themes that may be similar to those identified in the SWOT analysis with senior stakeholders, and b) themes that may be novel, different or elaborations of those identified in the SWOT analysis [7]. In addition, we wanted to seek provider recommendations to strengthen CAMHSS.

## Methods

### Study design

A qualitative exploratory study was conducted using focus group discussions (FGDs) and semi-structured individual interviews (SSIIs) in order to collect robust data to deepen the insight into the key issues that require attention in CAMHSS. Both SSIIs and FGDs were included to provide participating clinicians with flexible options to ensure that their clinical work would not be compromised. The same interview guide was used for SSIIs and FGDs. FGDs and SSIIs are useful in generating a rich understanding of participant experiences and beliefs [10].

### Study participants and setting

Purposive sampling was used to select CAMH professionals who were providing a direct service to children and adolescents with mental health problems and their families. Participants were selected from the health sector in the Western Cape. The Western Cape is divided into a number of urban (metropolitan) and rural districts. We aimed to include a wide range of service providers from all the urban health districts (Southern/Western substructure, Mitchell’s Plain/Klipfontein substructure, Tygerberg/Northern substructure, Khayelitsha/Eastern substructure), as well as rural districts (Cape Winelands, West Coast, Overberg, Eden and Central Karoo), and across all levels of care (primary, secondary and tertiary). Further detail about the health districts in the Western Cape including a map of districts and location of services is presented in two other publications [7, 11].

The Provincial Mental Health Directory [12] a list of all mental health professionals and their contact details, was used to identify the mental health service providers. Those who were not listed in the provincial directory were identified at facilities through facility managers. Participants were recruited both telephonically and via email.

### Data collection

Information sheets containing details about the study as well as consent forms were provided to invited staff, signed by the participants prior to the study, and were collected on the day of face-to-face interviews and/or focus group discussions. Data were collected between May and July 2017. SSIIs and FGDs were conducted by the primary researcher and two research assistants in a quiet, private space in a convenient location selected by each participant or group. The research assistants were mental health professionals with experience in conducting FGDs and SSIIs. All SSIIs and FGDs were audio recorded and field notes were taken during and after the interviews. The duration of FGDs and SSIIs ranged from 30 to 90 min.

An interview schedule was used with generic question covering the same topics and questions for both the FGDs and SSIIs. The discussions covered various topics exploring the lived experiences of providing child and adolescent mental health services, care pathways, intersectoral collaboration, barriers and facilitators to care, and recommendations on how to improve CAMHSS.

Member checking was done during the interview process and at the end of each SSII and FGD to optimise the trustworthiness of the findings. The interviewer summarised the key points that were made by the participants to validate the key issues that require attention in CAMHSS and then asked the participant(s) to confirm whether the information that they had given had been captured and interpreted accurately.

### Data analysis

Complete audio recordings were analysed using thematic analysis [13, 14] using the NVivo 11(QSR) qualitative data software package [15]. Data were coded, and codes were subsequently grouped into themes. The coded transcripts were analysed by running query reports, and primary document tables of the codes and themes were produced in order to explore the key issues.

To strengthen the trustworthiness of analysis, notes written by the three data collectors during FGDs and SSIIs were compared with the audio recordings to corroborate the findings of this study. Furthermore, to ensure robustness and replicability of the data, two researchers (SM and KJ) coded all the data independently. The two researchers met regularly to compare and discuss their findings until consensus was reached. No quantitative measure of agreement was calculated.

The research team (SM, PJdV, MS and KJ) collectively decided on representative quotes from the audio data. These were transcribed verbatim in NVivo.

## Results

### Characteristics of participants

A total of 46 mental health service providers across all districts in the Western Cape participated in this study. Participants included child & adolescent psychiatrists, general psychiatrists, medical officers (medically qualified professionals without formal qualifications in psychiatry), psychiatric registrars (medical doctors training in psychiatry), and mental health nurses. In total, eleven SSIIs and four FGDs were conducted. Table 1 provides a detailed summary of the study participants.

**Table 1:** Participants in the study of service providers

**An overview of participation**		
Total number of participants	n = 46	
Total number of focus group discussions (FGDs)	4 (rural = 1, urban = 3)	
Total number of semi-structured individual interviews (SSIIs)	11 (rural = 3, urban = 8)	
**Details of participants**		
	Child & adolescent psychiatrists = 4 Psychiatric registrars = 1 Medical officers = 1 General psychiatrists = 2 Mental health nurses = 38	
**Participation according to levels of care**		
Level of care	Primary	SSIIs = 3 FGDs = 4 (n = 35)
	Secondary	SSIIs = 4
	Tertiary	SSIIs = 4
**Participation according to geographic service areas**		
Geographical representation	City of Cape Town (urban)	SSIIs = 8 (child psychiatrist = 4, Psychiatric registrars = 1, Medical officers = 1, General psychiatrists = 2) FGD 1 (n = 4): Community mental health nurses FGS 2 (n = 4) Community mental health nurses FGD 3 (n = 7) Community mental health nurses
	Rural districts	SSIIs = 3 (Psychiatrist = 1, Community mental health nurses = 2) FGDs = 1 (n = 20) Community mental health nurses

The majority of study participants were mental health nurses. While this may be perceived as ‘disproportionate’, we deliberately oversampled nurses for two specific reasons: (1) mental health nurses represent the largest group in terms of the mental health workforce in the country, particularly at primary care, and (2) they are also typically the main gatekeepers for all mental health services in the country.

### Themes identified similar to those from the SWOT analysis [7]

The results of the FGDs and SSIIs based on identified themes are presented below. The themes identified that were similar to those from the SWOT analysis [7] are presented in Table 2.

**Table 2:** Themes identified in this study of grassroots service providers that overlapped with the SWOT analysis of senior stakeholders [7]

Overarching theme	Service provider themes (this study)	SWOT analysis Themes [7] (Mokitimi et al. 2019)	Illustrative examples/quotes
Lack of resources	Lack of CAMH infrastructure	Inadequate infrastructure and other resources	*“… I mean there’s no specific psychiatry ward; so many adolescent patients have to be admitted to normal ward with our other sick patients, which raise a lot more other issues. Even for the children, they also have to be admitted to the paeds (paediatric) ward with other sick children, and then you have this very disruptive kid amongst other sick children which is a challenge…”* Child & adolescent psychiatrist (secondary level, urban district)
	Heavy workload	Workload demands	*“… all the different clinics have their different challenges … and I'm gonna say that … to expect clinic sister to see children is … it's not … it's not realistic. They barely get to see their 50–60 adults, sometimes they’re 70 adults in the clinic per day … they barely get to see those patients the way they should be seen …”* District child & adolescent psychiatrist (secondary level, urban district)
	Lack of financing	Lack of dedicated funding for CAMH services	*“… mental health patients don't get the same quality of care as general patients do … even if you look at the departmental budgets … even psychiatry tends to get a smaller portion of budgets … so … you know … we are not able to provide services to exactly the standard that we would want …”* Medical officer (secondary level, urban district)
	Inequitable distribution of available resources	Inadequate and inequitable resource allocation	*“… there is inequity of service … which is very obvious … for example if I look at the size of my team here, the [tertiary CAMHS unit in one substructure] team is three times the size than is of [the other tertiary CAMHS unit in another substructure] … separate psychologist for each thing at outpatients, nurses seperate for each thing … it is really an imbalance in terms of distribution of resources … which is really challenging …”* Child & adolescent psychiatrist (tertiary level, urban district)
Lack of intersectoral collaboration	Silo working of the Departments of Health, Education and Social Development	Silo working of agencies	*“… big challenge is education, the department does not pick up these disorders soon enough. Social development is overloaded too …”* Child & adolescent psychiatrist (secondary level, urban district) *“… the social development system is overloaded and dysfunctional so that when a child has had a psychiatric experience, it becomes incredibly difficult to place them …”* Child & adolescent psychiatrist (tertiary level, urban district)
Limited training	Limited training	Limited training	*“The challenge is … the staff (in non-mental emergency inpatient units) is not trained to work with mentally ill patients and with adolescents … I feel for them because now they must deal with psychiatric patients …”* Mental health nurse (secondary level, urban district)
External contributing factors	Stigma	Societal stressors	*“… sometimes stigma is an issue, no parent wants their children to be labeled as a psychiatric patient…”* Child & adolescent psychiatrist (secondary level, urban district)

### Additional themes identified

Additional themes, not identified in the SWOT analysis [7] are summarised in Table 3, and described below with illustrative quotes.

**Table 3:** Additional themes from service providers on child and adolescent mental health services in the Western Cape

Overarching Theme	Examples
Lack of uniformity and consistency	Three specialist CAMH units work differently
Lack of support for staff	Lack of professional support Lack of emotional and moral support
Lack of acknowledgement of staff	Lack of acknowledgement for initiatives introduced by staff in CAMH services
Negative staff attitudes	Negative staff attitudes about seeing children and adolescents prevent them from providing good CAMH services
Health service innovations	Examples of innovative service provision by mental health nurses in primary healthcare clinics
External contributing factors	Poor socioeconomic circumstances Substance use

#### Lack of uniformity and consistency

Participants described that there was a lack of uniformity and consistency among the three tertiary specialist CAMH units in terms of the services offered, skills provided, structuring and in organisation of CAMHS. One specialised in autism, the other in neuropsychiatric conditions and the third in substance abuse and rehabilitation. Participants perceived specialist units to be implementing the national CAMH policy in their own way, including how they structured and organised CAMHS in their areas. Respondents further felt that there was no uniformity among the specialist CAMH units with regards to referral care pathways. The perception was that the one would accept referrals only from medical professionals, while the others accepted referrals from any professionals. Service providers felt that this perceived lack of uniformity created confusion not only to them, but also to service users when they move between the catchment areas for these units.

“The units are running very inefficiently. For example, very few people know about what’s happening where. Thus, too many things in parallel and in isolation. Service offerings are not consistent in terms of processes, and resource allocation is imbalanced, which leads to certain services can only be offered at certain places.”

Child & adolescent psychiatrist (tertiary level, urban district)

#### Lack of support for staff

Participants felt that there was a lack of professional and emotional support for staff. For instance, they described having to pay for their own private supervision and emotional/therapeutic support. Community mental health providers felt that they did not receive support from the specialists in the specialist CAMH units, especially with difficult cases that they manage at primary level. Mental health providers in rural areas felt that there was very limited interaction between them and specialist CAMH team members.

“… very little liaison with tertiary levels so we don’t really have much support from the tertiary [services] …”

Child & adolescent psychiatrist (secondary level, rural district)

“… It’s an incredibly stressful discipline, which makes you want to cry, kill people, and therefore … needs to be a support system for containment and there’s no budget for that … so we occasionally end up having to support ourselves in the group meetings. I myself run group support but I myself need one of those so I have to pay for it, but not everyone can afford that …”

Child & adolescent psychiatrist (tertiary level, urban district)

#### Lack of acknowledgement of staff

A number of mental health nurses at primary healthcare facilities felt that they had developed potential CAMH service models that were working well in their facilities, and that these service models could be rolled out to other facilities. The challenge was that they felt facility managers and DoH provincial planners did not notice and did not acknowledge them. Instead, the DoH provincial planners continued to invent models that were not working, according to the providers.

“… we don't really get incentives for services ... at least that is what would make what we do worth it ... it would be very nice and very grateful and appreciated if management comes and say ... Mr ... now you started the Tuesday [clinic] ... and I can see you went through all this lengths to get it established and running ... patients are happy and we can see the kids are getting back to school early ... thank you for what you put in...”

Mental health nurse (primary level, urban district)

“… we sit in mental health forums ... and we present in the forums that [is what] we do because ... these people in the forums are talking stuff that we are already busy doing ... and they're still trying to figure out how they're going to get us right ... but it is there and it's already been done...”

Mental health nurse (primary level, urban district)

#### Negative staff attitudes towards seeing children and adolescents

Mental health nurses at primary healthcare facilities felt that the negative attitudes of their fellow mental healthcare providers towards CAMHS was a problem. A number of staff said that they worked hard to provide the best possible services for their patients under difficult and resource-constrained conditions. They took initiative to create structures within their facilities, within their specialist services and with other non-specialists, and managed to provide CAMHS that at least met the needs of their users. The majority of mental health nurses at primary level, however, felt overwhelmed and unable to provide CAMHS in their settings. The few mental health nurses who felt that they could provide good CAMHS said that their colleagues need to change their negative attitudes towards CAMHS and be open to restructuring their services in order to accommodate children and adolescents. They acknowledged that they also experienced challenges but still felt strongly that it was possible to provide good CAMHS if negative attitudes can change.

“I think the services is great ... because we as clinicians we tend to complain that we can't take this on board ... we have too much of adult psychiatric patients ... and whatever ... but I think if we have proper systems in place ... like putting the children on a specific day it will actually make your work routine and work life much more easier ... I think other clinicians are not realising that, and that's why they ... they're actually ... I feel like kind of negative to take this type of systems on board and dismissing it as ‘I don't have time’ ... but not really seeing that it will actually spare time … I would recommend you know ... other mental health clinicians to also think positively about the outcome of having a child clinic at the day hospital clinic setting...”

Mental health nurse (primary level, urban district)

#### Health service innovations

A few mental health nurses at PHC facilities shared their innovations for providing ideal effective CAMHS despite all their challenges with resources. For example, there were facilities that provided stand-alone CAMHS. In some facilities, a specific day of the week was set aside as a CAMH clinic, or specific afternoons were set aside for children and adolescents only. Children and adolescent were not mixed with adult psychiatric patients. Mental health nurses coordinated CAMHS within the facilities in such a way that they were prioritised in all departments (administration, pharmacy and general assessments) so that they did not miss out at school. Their files were kept in a mental health section.

“… otherwise I did do the talks at schools, so even the teachers know what type of children to refer to me … I do ADHD outreaches … I am working in a multidisciplinary team with the MOs … I have a child- friendly service … I don’t want them to wait among adults in a day hospital setting … so I take the folders straight to the doctor and the doctor will do the work-up…”

Mental health nurse (primary level, urban district))

“… the facility managers can now see that they need to have a child- friendly services … but that was only now in the Desmond Tutu youth-friendly initiative … they launched the youth-friendly services last week Friday, and we started already in January … we were like ahead of them … we were almost like the main leaders …”

Mental health nurse (primary level, urban district)

#### External factors contributing to poor child and adolescent mental health

HIV/AIDS, substance use and stigma were identified by providers as external factors contributing to poor child and adolescent mental health. Unresolved social circumstances create a vicious cycle as children keep going back to the same circumstances. Stigma impacts on help-seeking behaviour for CAMH, and drug use is a problem for children and adolescents who are susceptible to mental illness.

“… because we’re treating the same people…they go back to the same social circumstances…nothing has changed…it becomes a vicious cycle …”

Mental health nurse (primary level, urban district))

“… substances is a big problem, and substances can’t be dealt with at the *Cape Flats, it needs to be dealt with a higher level …”

Child & adolescent psychiatrist (secondary level, urban district)

* geographical part of the City of Cape Town

### Recommendations to improve child and adolescent mental health services

Participants made eight recommendations to improve CAMHS in the Western Cape, as outlined in Table 4. These included increasing CAMH staffing, providing dedicated CAMHS at secondary care and creation of child-friendly infrastructure at primary care, review of current requirements for high volume output rather than quality service delivery, formalisation of intersectoral collaboration, increased learning opportunities for trainees, employment of a ‘lead’ professional for CAMH in the province, providing more support for staff, and acknowledging staff initiatives in CAMHS.

**Table 4:** Service provider recommendations to strengthen child and adolescent mental health services

1. Increase CAMH staffing
2. Provide dedicated child and adolescent mental health services at level 2 (secondary care) and child-friendly infrastructure at level 1 (primary care)
3. Review current service focus on number of patients seen versus quality of care provided to children
4. Formalise intersectoral collaborations
5. Increase learning opportunities for trainees
6. Employ a lead professional for CAMH in the province
7. Increase support for staff
8. Acknowledgement of staff initiatives

#### Increase child adolescent mental health staffing

CAMH providers recommended that each primary healthcare facility should be staffed with at least two mental health nurses, an intern psychologist or clinical psychologist, an occupational therapist, a psychiatric registrar and a social worker. At secondary level there should be specialist CAMH providers and mental health multidisciplinary professional for CAMHS.

Providers emphasised that there should also be equitable distribution of human resources across the three specialist CAMH units and across the three sub-structures.

“… specialist CAMH professionals should be allocated at level 1 [primary healthcare level] who will engage with children on their level. There should be clinical psychologists at every CHC [community health centre]. There should be a tertiary CAMHS outreach team that goes to see children and adolescents at level 1.”

Mental health nurse (primary level, urban district)

#### Provide dedicated child and adolescent mental health services at secondary care level (level 2) and child-friendly infrastructure at level 1

Providers recommended that there should be inpatient and outpatient mental health units exclusively for children and adolescents at secondary care level. These units should have their own multidisciplinary teams and child-friendly assessment and intervention tools. At primary level all services should be provided with child-friendly assessment tools in order to engage therapeutically with children and adolescents.

“I think there should be something between us and tertiary, for children specifically...Like I said maybe beds in the district level where there could be a team, a psychiatric team that are seeing children there, then if the cases are complicated and need tertiary level then, they come from there to this level, because now there is nothing between us and tertiary, for children ...”

Mental health nurse (primary level, urban district))

“… the environment at clinic levels should be I feel like … colourful … not this grey, cream or whatever … but colourful ... attracting this child to say I’m feeling like I’m in a space where I can express myself and say anything … that will be ideal…”

Mental health nurse (primary level, urban district)

“… I can just agree with others with the environment itself … when I started there I sort of asked for these things ... chairs for the little ones … pictures … then I decided to buy my own just to have it there ... they [children] run to these chairs … it makes it so much nicer … that is actually important for a child to feel comfortable in that area …”

Mental health nurse (primary level, urban district)

#### Review current service focus on number of patients seen versus quality of care provided to children

Providers felt that they need to be relieved from the current requirements of having to see a set number of patients per day and capturing these statistics. They said that the administrative duties add to their workload, and yet it does not reflect on the amount of work done in a day. They felt that the focus should be shifted to quality of service for children and adolescents rather than just the quantity.

“… because now we must fight to keep those stats … basically it’s just totals based … I won’t be able to give that quality of therapy … what is the point of seeing the child for five minutes because you wanna build stats, then you’re not really doing anything…not providing therapy…”

Mental health nurse (primary level, urban district)

#### Formalise intersectoral collaborations

Providers recommended that collaborations between the DoH, WCED, WCDSD and the Department of Justice should be formalised at top management level in the province. Each department should be clear on their roles and responsibilities for CAMHS and referral care pathways.

“I feel that mental healthcare services can’t stand on its own…Department of Health, it needs the Department of Social Services intimately linked with it ... because it is a struggle when you find a child who is being abused…it needs … it’s not a simple process to...you know…access those services…”

Psychiatric registrar (tertiary level, urban district)

#### Increase learning opportunities for trainees

Mental health providers felt that trainees should not be counted as part of the workforce during their academic placement in the facilities, because the workload deprives them of the opportunity to learn. Trainees should be afforded the opportunity to learn in the field.

“… but what I’m saying is that perhaps six months is not enough… perhaps there needs to be a structural change in the Department [of Psychiatry] so that they don’t see registrars as gap fillers but as students, as people who need skills rather than human resource gap fillers… so there’s also to be mindful that these people are actually here to learn and not to fill the gap of lack of human resources…”

Psychiatric registrar (tertiary level, urban district)

#### Employ a lead professional for child and adolescent mental health in the province

Participants recommended that a senior lead professional post for CAMHS should be created as part of the senior management structure in the province. The ‘CAMHS lead’ could coordinate services in the three substructures, in rural districts and across sectors. Such a post should ensure that all three specialist CAMH units are uniform and consistent in service delivery, for instance through a line management function to the heads of the CAMHS units. In addition, the CAMHS lead professional will oversee all levels of care in the province to ensure equitable distribution of services and resources across the province.

“My ideal is, there must be someone higher up in charge of child and adolescent psychiatry. I really feel that's what's lacking, to kind of draw people together, to draw the three big units together and see it from the top…”

Child & adolescent psychiatrist (tertiary level, urban district)

#### Increase support for staff

Participants felt that CAMH specialists should be more visible in rural areas and in primary healthcare facilities to support staff. For instance, they could offer outreach services to primary healthcare by doing consultations with patients and providing supervision to primary healthcare staff. Staff also felt that they need to be supported by the provincial Department of Health to access clinical supervision, mentoring and emotional support.

“Tertiary institutions need to support us. They should have something like outpatient clinic for our difficult cases.”

Mental health nurse (primary level, urban district)

#### Acknowledgement of staff initiatives

Providers felt that their efforts and initiatives should be acknowledged, and that the Department of Health should consult with them to learn about their grassroots initiatives and innovations in CAMH. They felt that some of these initiatives may have potential for implementation in other parts of the provincial CAMHS.

“… I do think with the little that we have and that we do… we do a difference in one or two… and I must add on there’s not just the numbers that go up, but the successful cases that go up…You’re running your own clinic… but basically everything is your own… I feel that community mental health nursing is gonna grow to such a level that if they [management] don’t receive that [acknowledgement of staff initiatives], they’re [community mental health nurses] gonna look for something that is gonna satisfy them…”

Mental health nurse (primary level, urban district)

## Discussion

Given the limited data on the views of service providers at grassroots level in the Western Cape about CAMHSS, this study sought the perspectives of a range of frontline service providers across all levels of care and across all health districts in the province. We conducted face-to-face semi-structured individual interviews and focus group discussions and asked clinical staff about their experiences on CAMHSS, and their recommendations for future strategies that might strengthen CAMHSS. The themes that emerged not only highlighted challenges with both hardware and software elements of the health systems for child and adolescent mental health, but also pointed to a number of potential solutions to strengthen CAMHS in the province.

In terms of hardware elements, participants expressed challenges with the lack and inequitable distribution of CAMH resources, heavy workload and the lack of financing. These findings show a lack of progress in the Western Cape, given that there have been more than 10 years since the last observations by Dawes [16], the distribution of resources remains unchanged.The findings in this study therefore represent a call to action for government to implement appropriate actions to strengthen CAMHSS in the province and country. Our findings also highlight the lack of infrastructure and coordination, and of governance and leadership in CAMHS, thereby suggesting that services are currently unresponsive to the needs of children and adolescents with mental health problems, and unresponsive to the requirements of service providers at grassroots level. A particular point was made about the lack of child and adolescent mental health services at secondary care (level 2), where primary care staff perceived there to be nothing in between them and specialist CAMH (level 3/tertiary services), and which they found difficult to access.

The software-related challenges identified included a lack of intersectoral collaboration, inadequate training on CAMHS, lack of support for staff, lack of acknowledgement of staff initiatives, negative attitudes of some staff towards the mental health of children and adolescents, and comments on external contributing factors such as stigma, socio-economic challenges and substance use in local communities. These issues suggest gaps in relationships between the provincial leadership, managers, planners and policymakers on the one hand, and service providers at grassroots level on the other. It was clear from the qualitative data that this perceived disconnect had a negative impact on the morale of service providers, risking the quality of service provision for users.

We also observed interactions between hardware and software elements, and how they impact on each other. For instance, service providers expressed feeling demoralised and inadequate in rendering effective services because of the structural challenges and lack of good leadership and governance in CAMHS. If these challenges are left unresolved, they will continue to threaten job satisfaction and sustainability for service providers [17]. The capacity for CAMHS may be further reduced if and when service providers become intolerant of continuing to work under unfavourable conditions and leave the service.

We were, however, encouraged by the recommendations made by the participants to strengthen CAMHS. With the exception of hardware recommendations to increase staffing and develop dedicated CAMHS at secondary care level, all other recommendations appeared to be software elements that could be implemented almost immediately. The example of one facility where staff introduced innovations to provide a responsive service despite their hardware challenges, exemplified the importance of software elements in service strengthening, i.e. leadership, positive attitudes, acknowledgement and recognition of staff initiative. Chunharas and Davies [18] pointed out that good leadership at different levels can strengthen a health system. This requires having a vision, setting priorities and mobilising stakeholders and resources to achieve the goals. However, without support from the leadership, staff morale can be impacted negatively, and grassroots innovations can fail, instead of being celebrated and rolled out to other settings. Our findings therefore identified a strong message about the potential to use the local expertise and innovation of mental healthcare staff (often mental health nurses) at grassroots level to strengthen CAMHSS.

The recommendations call for a review of the current structure and service delivery model for CAMHSS. The ideal structure for CAMHSS as recommended by service providers should ensure: 1) proper coordination of the services at top provincial level, within the DoH and with other departments, and down to the primary care level, 2) adequate capacity for CAMH across all levels, 3) collaboration between senior and grassroots level stakeholders, and 4) reasonable and relevant performance requirements for service providers. They also recommended provision of good leadership and governance at provincial level.

Many of the themes identified in this study were similar to those identified in the situational analysis [9].The findings of these three investigations validates and reinforces the significance of the challenges for child and adolescent mental health services in the Western Cape. Our findings also concur with broader South African and international literature. For instance, a qualitative study that explored multistakeholders’ perceptions of CAMHSS in another South African province (KwaZulu-Natal) found that there was a shortage of CAMH resources (human and infrastructure) resulting in service providers being overwhelmed with their workload, inadequate CAMH training for non-specialists, lack of a coordinated system of CAMH, and stigmatisation of mental illness in children and adolescents [19]. In an international systematic review that explored primary care practitioners’ perceptions of the barriers to the effective management of CAMH problems, the authors found lack of staff training, lack of prioritisation of mental health problems, lack of resources, and family issues as key barriers [20]. Our findings are therefore likely not only to be applicable in the Western Cape and in South Africa, but also in other settings, particularly in LMIC.

## Limitations of the study

We acknowledge a number of potential limitations of the findings in this study. First, data were collected in 2017 and therefore there may have been some hardware or software changes in CAMHS after the study. However, as practitioners within the system under investigation we have not observed any significant changes that would invalidate the observations made here. Our findings are also the most comprehensive exploration of service provider perspectives in the province to date. Second, given the qualitative nature of the work, we acknowledge the possibility that we may have missed important themes in this study. However, to mitigate against that, data analysis was performed by two independent raters (one of whom had never worked in the CAMH system) to ensure robustness. Third, participants did not include all professional groups (e.g. occupational therapists, speech and language therapists or psychologists) or adequate rural representation. We acknowledge that the full range of disciplines and allied health professionals are crucial to comprehensive CAMHS and that additional themes may have emerged through their participation. Similarly, rural CAMHS requires further exploration. It would therefore be important to include these groups in future studies. It would also have been important to understand the root causes of the negative attitudes of mental healthcare workers towards CAMHS, a theme not explored in this study. Literature shows that lack of knowledge, stigma and lack of training in CAMH are some of the contributing factors to negative staff attitudes toward child and adolescent mental health [21], and these contributing factors were also identified in this study. Given the oversampling of nurses (as outlined in the methods section), we acknowledge that some of the themes may have general applicability beyond the care of the children with mental health problems and may have related to challenges in broader community-based nurse practices. However, the focus of our work was on CAMH, and we were therefore cautious not to overinterpret our findings. Finally, we acknowledge that the voices of children and families were not included in this study. However, given the importance of their voices, we have opted to perform a separate investigation dedicated to the perspectives of families and CAMH service users.

### A comment on reflexivity

We acknowledge the position of the author team in terms of the study and the intent of data analysis. In particular, we acknowledge that the first author was a mental health nurse by profession, with more than 20 years of experience in adult and child and adolescent psychiatry in the Western Cape Province of South Africa. We therefore acknowledge that prior experience may have biased our exploration of the themes and issues in this study. However, we believe that this experience and understanding of CAMHS also allowed us to provide a more nuanced and deep analysis of the data. As outlined in the methods, we consciously aimed to collect data in a robust and replicable manner, sought independent supervision throughout the study, and ensured inclusion of non-clinical second coders who had not worked in the clinical system for all qualitative data analysis.

We therefore trust that the combination of our conscious and unconscious contributions to this study provided a contemporary evidence-base that may help to strengthen CAMH services and systems in the Western Cape Province of South Africa and beyond.

### Relevance of findings to other low and middle-income-countries

Whilst the specific focus of our qualitative study was the Western Cape of South Africa, our context (including limited resources, poor implementation of policies, negative attitudes towards CAMH) is very similar to that of most other low- and middle-income countries. We therefore believe that our findings may also have a direct or indirect relevance not only to other South African provinces, but also to other LMICs.

## Conclusions

These findings provided insight into the perceptions of service providers at grassroots level about the current state of CAMHSS in the Western Cape province of South Africa. The challenges identified here highlighted the hardware and software weaknesses in CAMHSS. The observations and recommendations made are directly relevant to system strengthening in the Western Cape but we propose that the themes identified may be equally relevant to other provinces and other LMICs. We sincerely hope that our findings will provide service developers, funders and policymakers with an evidence-base that can be used to strengthen CAMHS, ultimately to ensure that we meet the needs of all our service users.

## Data Availability

The dataset used and analysed during this study is available from the authors on reasonable request.

## Abbreviations

CAMH: Child and adolescent mental health
CAMHS: Child and adolescent mental health services
CAMHSS: Child and adolescent mental health services and systems
CHC: Community health clinic
DoH: Department of Health
LMIC: Low- and middle-income countries
PHC: Primary health care
WCDSD: Western Cape Department of Social Development
WCED: Western Cape Education Department
WHO: World Health Organization
